# Enhancing the efficacy of VEGF inhibitors by co-inhibition of HIF in the treatment of glioblastoma

**DOI:** 10.1007/s10495-026-02275-5

**Published:** 2026-01-23

**Authors:** Emirhan Harbi, Yasemin Yozgat Byrne, Hamza Ugur Bozbey, Didem Tastekin, Oral Oncul, Soha Hosseiny, Duha Yahya, Ozcan Yildiz, Murat Erdogan, Abdul Kadir Slocum, Christopher E. Mason, Michael Aschner

**Affiliations:** 1 Department of Molecular Biology and Genetics, Faculty of Engineering and Natural Sciences, Bahcesehir University, 34353 Istanbul, Turkey; 2 Computational Biology and Molecular Simulations Laboratory (HITMER), Department of Biophysics, School of Medicine, Bahcesehir University, 34734 Istanbul, Turkey; 3 Research Institute for Health Sciences and Technologies (SABITA), Cancer Research Center, Istanbul Medipol University, 34956 Istanbul, Turkey; 4 Department of Clinical Oncology, Institute of Oncology, Istanbul University, 34093 Istanbul, Turkey; 5 Biruni University Research Center (B@MER), Biruni University, 34015 Istanbul, Turkey; 6 Department of Medical Oncology, Biruni University School of Medicine, Istanbul, Turkey; 7 Department of Infectious Diseases and Clinical Microbiology, Internal Medicine, Istanbul Faculty of Medicine, Istanbul University, 34093 Istanbul, Turkey; 8 Department of Molecular Biology, Uskudar University, Istanbul, Turkey; 9 Basaksehir Cam and Sakura City Hospital, Istanbul, Turkey; 10 Department of Orthopaedics and Traumatology Surgery, Medical Faculty, Ondokuz Mayıs University, Samsun, Turkey; 11 Medical Oncology, ChemoThermia Oncology Center, 34365 Istanbul, Turkey; 12 Department of Physiology and Biophysics, Weill Cornell Medicine, New York, NY 10065 USA; 13 Department of Molecular Pharmacology, Albert Einstein College of Medicine, 1300 Morris Park Avenue, Bronx, NY 10461 USA

**Keywords:** Glioblastoma, HIF, VEGF, Treatment resistance, Molecular biology of glioblastoma

## Abstract

Glioblastoma is the most aggressive and most common grade 4 tumor of the central nervous system (CNS). Despite standard treatments such as surgical resection and chemoradiotherapy, overall survival (OS) usually does not exceed 14–16 months in clinical trials, and no improvement in OS has been demonstrated even with the use of vascular endothelial growth factor A (VEGFA) inhibitors such as bevacizumab. In response to radiotherapy, hypoxia-inducible factor (HIF) stabilization leads to activation of alternative pro-angiogenic pathways, increasing VEGF expression and tumor angiogenesis. Several clinical trials evaluating HIF-2α inhibitors as monotherapy in the absence of concurrent VEGF inhibition, have similarly failed to demonstrate a significant improvement in OS outcomes. This review provides a perspective on the combined use of VEGF and HIF inhibitors, and provides an insight into future studies.

## Introduction

Glioblastoma remains the most aggressive and most common treatment-resistant primary central nervous system (CNS) tumor, characterized by a highly vascularized and hypoxic microenvironment [1, 2]. Standard treatment involves tumor resection followed by chemoradiotherapy with temozolomide (TMZ) and subsequent adjuvant TMZ administration; despite this, glioblastoma patients have an overall survival (OS) of approximately 14–16 months in clinical trials [3]. One of the key features of glioblastoma is abnormal angiogenesis, which promotes the formation of dysfunctional, leaky blood vessels that contribute to tumor progression, predominantly through vascular endothelial growth factor (VEGF), and therapeutic resistance [4]. Even with the administration of VEGF inhibitors such as bevacizumab, which aim to reduce vascularization and peritumoral edema, no significant improvement in OS has been demonstrated in glioblastoma [5]. Hypoxia-inducible factor (HIF) has a significant effect in mediating adaptive resistance to VEGF inhibition [6]. Under hypoxic conditions, HIF-1α and HIF-2α are stabilized, leading to activation of various pro-survival pathways through angiogenesis and invasion. VEGF signaling inhibition exacerbates hypoxia within the tumor microenvironment (TME), further stabilizes HIFs, and induces the expression of compensatory angiogenic factors such as platelet-derived growth factor (PDGF), fibroblast growth factor (FGF), and stromal-derived factor-1 (SDF-1) [7]. This hypoxia-induced resistance significantly attenuates the efficacy of VEGF-targeted therapies and indicates the potential for evaluating combination strategies that concurrently inhibit both VEGF and HIF signaling pathways. The combination of VEGF inhibitors and HIF blockade in glioblastoma has not yet been comprehensively evaluated in any clinical trial; it is a hypothesis supported only by preclinical data and known mechanisms, and more comprehensive clinical studies are needed in this area.

Concurrent inhibition of VEGF and HIF signaling pathways may represent a promising therapeutic strategy to overcome treatment resistance in glioblastoma. Dysregulation of HIFs induces angiogenesis by increasing VEGF expression, while abnormal activation of STAT3, and NF-κB pathways increases tumor-associated inflammation. Recent preclinical trials have demonstrated that pharmacologic HIF inhibition enhanced the efficacy of VEGF inhibitors by suppressing the compensatory anti-angiogenic response. HIF-2α-targeting agents, such as Belzutifan, have demonstrated efficacy in disrupting hypoxia-induced oncogenic signaling. By blocking HIF-mediated gene expression, these inhibitors not only suppress alternative angiogenic pathways, but also reduce the expression of key survival factors that promote tumor proliferation and invasion [8].

### Molecular characteristics of glioblastoma: A summary

The 2021 World Health Organization (WHO) classification of CNS tumors introduced important changes in the classification of glioblastomas [9]. This new classification emphasizes both histopathological and molecular features to define glioblastomas [10, 11]. Specifically, glioblastomas are presently classified as IDH-wild type gliomas, which may show histologic features such as microvascular proliferation, and necrosis, or molecular alterations such as TERT promoter mutation, EGFRvIII amplification, and gain of chromosome 7 concurrent with loss of chromosome 10 (+ 7/− 10). The O^6^-methylguanine-DNA methyltransferase (MGMT) promoter methylation has an important role in determining treatment resistance, especially to alkylating agents such as TMZ.

### HIF pathway and RT utilization

Elucidating the role of the HIF signaling pathway in glioblastoma and its interaction with RT holds significant potential for informing the development of future therapeutic strategies [12, 13]. In hypoxic conditions, the HIF-α subunits stabilize and translocate to the nucleus where they dimerize with HIF-β subunits to activate transcription of survival-related genes. In addition, ^18^F-FMISO PET can be used to monitor hypoxic and vascular regions in gliomas [14–16]. HIF-2α is a critical regulator in glioblastoma, contributing to tumor progression and the maintenance of cancer stem cell (CSC) populations [17, 18]. Recent advances in the field have led to the development of HIF-2α inhibitors such as Belzutifan. Belzutifan has shown efficacy in paragangliomas, and Pacak-Zhuang and Von Hippel-Lindau (VHL) syndromes, characterized by polycythemia. The U.S. Food and Drug Administration (FDA) approved Belzutifan in VHL-associated tumors on August 13, 2021 [19–21]. Its application in the treatment of glioblastoma enhances existing therapies by disrupting HIF mechanisms that promote tumor progression. Thus, targeting HIF-2α could improve the efficacy of existing therapies by disrupting the ability of the tumor to develop under hypoxic conditions.

### VEGF pathway in glioblastoma

**Fig. 1:**
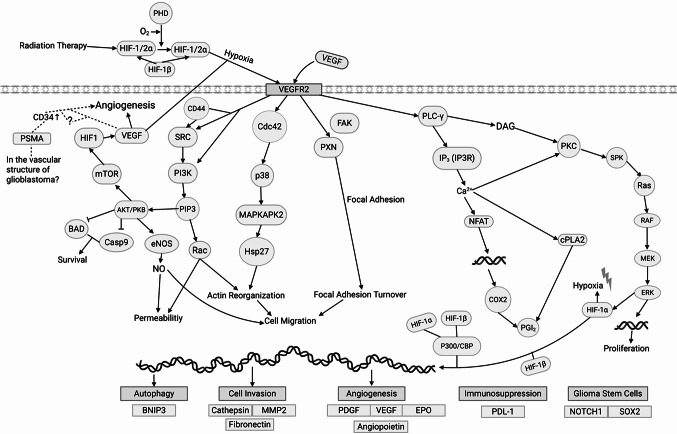
VEGFR2 pathways in glioblastoma

VEGF is a key regulator of endothelial cell proliferation, chemotaxis, survival, and blood-brain barrier (BBB) permeability [22]. Specifically, the VEGF-A isoform is the most important ligand for VEGF receptors, and predominantly mediates angiogenic effects, including VEGF receptor 2 (VEGFR2) in glioblastoma [23–25]. VEGF promotes the formation of new blood vessels that supply glioblastoma with oxygen and nutrients, facilitating its rapid growth and progression [26]. This angiogenic activity is a hallmark of glioblastoma and an important factor in its poor prognosis. The signaling pathway and molecular interactions involving VEGFR2 in glioblastoma are shown in Fig. 1.

Figure 1 Vascular endothelial growth factor receptor 2 (VEGFR2)-mediated cellular signaling pathways were demonstrated in glioblastoma. Under hypoxic conditions, the activation of HIF-1α and HIF-1β increases VEGF synthesis, and this ligand binds to VEGFR2, inducing several cellular mechanisms [27, 28]. VEGFR2 activation stimulates major pathways such as PI3K/AKT/mTOR, MAPK/ERK, PLCγ/PKC, and FAK, supporting cell survival, proliferation, migration, adhesion, and angiogenesis [29]. The PI3K/AKT pathway regulates vascular permeability by increasing nitric oxide production via eNOS, while MAPKAPK2 and Hsp27 play a role in cell migration and actin reorganization. PLCγ activation results in the activation of PKC, Ras, and ERK pathways, accelerating cell proliferation. In addition, HIF-1 activation contributes to autophagy, cell invasion, angiogenesis, immune suppression, and glioma stem cell maintenance by regulating the expression of genes such as BNIP3, MMP2, VEGF, PDGF, PDL-1, NOTCH1, and SOX2, respectively. PSMA plays a role in neovascularization with CD34, but the possibility that HIF activation could increase VEGF expression via PSMA is still theoretical and subject to research [30]. VEGFR2-mediated signaling networks play a central role in vascular formation, tumor growth, and treatment resistance mechanisms, which form the basis of the aggressive phenotype of glioblastoma [31, 32].

In addition to its role in angiogenesis, VEGF affects cell motility in glioblastoma. VEGF stimulates the motility of glioblastoma cells and contributes to their invasive behavior. However, the effect of VEGF on cell proliferation in glioblastoma is more complex. While VEGF can stimulate proliferation in certain cell types, such as glioma stem cells (GSCs), its effect on established glioblastoma cell lines is not yet fully understood [33]. RT can upregulate VEGF expression and enhance cell motility, but not proliferation [34–36].

RT has been shown to induce HIF activation [37, 38]. Normoxically, the HIF-1α subunit was hydroxylated by prolyl hydroxylase enzymes and degraded by the von Hippel–Lindau (VHL) ubiquitin-proteasome [21, 39, 40]. However, during hypoxia this hydroxylation process was inhibited, leading to stabilization and accumulation of HIF-1α [6]. Stabilized HIF-1α is transported to the nucleus, where it dimerizes with HIF-1β and then binds to hypoxia-responsive elements (HREs) in target gene promoters, activating transcription of genes involved in angiogenesis and survival pathways [41–43]. However, ionizing radiation used in RT can induce HIF-1α expression even in the presence of normal oxygen levels [38, 44]. Radiation-induced HIF-1α activation regulates the functional interaction between HIF-1α and the DNA-dependent protein kinase catalytic subunit (DNA-PKcs), which is mediated by hypoxia, by enhancing DNA damage response (DDR) pathways [45–47]. Consequently, the elevated expression of VEGF in glioblastoma enhances the already existing angiogenesis and contributes to the radioresistance of glioblastoma by facilitating metabolic reprogramming to promote tumor survival.

From a clinical perspective, RT remains a cornerstone in the treatment of glioblastoma; however, while it induces tumor cell apoptosis, it concurrently activates or amplifies HIF signaling pathways. Activation of hypoxia-inducible factors (HIFs) amplifies the already elevated expression of VEGF through the VEGFR2 signaling pathway in glioblastoma. Since the standard RT regimen has been administered without concurrent HIF inhibition since 2005, it is plausible that this has contributed to persistently elevated VEGF expression levels. This may, in turn, diminish the therapeutic efficacy of bevacizumab, which is often employed to counteract severe angiogenesis. The limited impact of bevacizumab under these conditions highlights a critical area of investigation: the integration of HIF inhibitors into RT-based treatment protocols. Given the central role of HIFs in tumor hypoxia and angiogenesis, such inhibitors hold potential for broad applicability across various cancer types treated with RT. The one exception is that patients with glioblastoma are usually elderly and may not respond well to hypoxic conditions, which may limit the use of HIF inhibitors because of side effects such as the development of congestive heart failure. However, the combined use of VEGFR2 inhibitors and agents targeting the HIF pathway may offer a synergistic therapeutic strategy to effectively suppress angiogenesis, thereby potentially improving OS in patients with glioblastoma.

### HIF and VEGF inhibitors for glioblastoma

A recent phase II study investigated PT-2385 (NCT03216499), an oral HIF-2α inhibitor, in patients with recurrent glioblastoma [48]. This phase II study included 24 patients who experienced the first recurrence of glioblastoma following standard chemoradiotherapy treatment. Participants received PT-2385 at a dose of 800 mg twice daily. The results showed that PT-2385 was well tolerated in patients. Grade 3 drug-related side effects included hypoxia (observed in two patients), anemia, hyperglycemia, hyponatremia, and lymphopenia. Despite an acceptable safety profile, no objective radiographic responses were observed. The median progression-free survival (PFS) was 1.8 months. Specifically, patients with higher systemic exposure to PT-2385 experienced a longer median PFS of 6.7 months, suggesting a possible exposure-response relationship. However, these patients had not been treated with VEGF inhibitors and no significant alterations in serum VEGF concentrations were observed during PT-2385 treatment.

In the LITESPARK001 study conducted by Strowd et al., no improvement was observed in the outcomes of single-agent Belzutifan in glioblastoma [49]. Only 2 of the 25 patients in the study showed disease stabilization, while 23 developed tumor progression. The poor clinical outcome observed in this study clearly demonstrates that Belzutifan is insufficient when used alone as a single agent.

Patients receiving RT have shown HIF activation and increased VEGF expression; however, standard treatment does not include a VEGF inhibitor, and VEGF inhibitors are not commonly used in the absence of a HIF-2α inhibitor, because they do not show efficacy. In this clinical trial, the sequential administration of a VEGF inhibitor such as bevacizumab, or a multi-kinase inhibitor like sunitinib following treatment with the HIF-2α inhibitor PT-2385, was proposed as a potential therapeutic strategy. This combination may overcome resistance in glioblastoma cases that have previously shown limited responsiveness to VEGF-targeted therapies [48]. A summary of HIF inhibitors is shown in Table-1.

Rapisarda et al. used bevacizumab and the HIF-1α inhibitor Topotecan together in 10 mice xenograft models created with U251 glioblastoma cells [50]. It was observed that bevacizumab treatment alone reduced microvascular density, increased hypoxia, but failed to induce apoptosis; it even increased HIF-1-dependent gene expression. In contrast, in the treatment group supplemented with topotecan, tumor growth was significantly suppressed, HIF-1 transcriptional activity was inhibited, cell proliferation decreased, and apoptosis increased. This study supports that the hypoxic signal developing as an adaptive response to anti-VEGF treatment is mediated through HIF-1 and that simultaneous inhibition of HIF-1 may enhance treatment efficacy.

**Table 1:** HIF inhibitors

HIF inhibitor	Mechanism of action	Clinical status
Belzutifan (PT2977, MK-6482) (NCT02974738), [51], [52]	HIF-2α inhibitor	FDA-approved (VHL-associated tumors), phase I solid tumors
PT-2385 [48], [53]	HIF-2α inhibitor	Phase I/II
Acriflavine [54], [55], [56]	Inhibits HIF-1 dimerization	Preclinical
PX-478 [57], [58]	Reduces HIF-1α protein levels	Preclinical
YC-1, echinomycin, and topotecan [59]	Inhibits HIF-1α transcriptional activity	Preclinical for glioblastoma
BAY 87-2243 [60]	Suppresses HIF-1α accumulation	Preclinical

A major clinical placebo-controlled phase III trial investigated the addition of bevacizumab, a monoclonal antibody (mAb) targeting VEGF-A, to the standard treatment regimen of RT and TMZ in patients with newly diagnosed glioblastoma [5]. The study included 637 patients, and evaluated improvements in OS, and PFS. The results showed that there was no significant difference in OS between the Bevacizumab group and the placebo group, with median OS times of 15.7 and 16.1 months, respectively. However, PFS was longer in the bevacizumab group (10.7 months) than in the placebo group (7.3 months). Specifically, patients receiving bevacizumab had increased rates of hypertension, thromboembolic events, intestinal perforation, and neutropenia. In addition, these patients showed more severe symptoms, reduced quality of life, and decline in neurocognitive functioning over time [61]. Similar studies with diverse VEGF inhibitors have generally corroborated this conclusion, namely, that current VEGF inhibitors alone are insufficient to fully suppress angiogenesis in glioblastoma, and their use has not been associated with a significant improvement in OS [62, 63]. A summary of VEGF inhibitors is shown in Table-2.

**Table 2:** VEGF inhibitors

VEGF inhibitor	Mechanism of action	Clinical status
Bevacizumab [5]	VEGFA inhibitor	Phase I/II
Sunitinib [64], [65]	Multi-targeted tyrosine kinase inhibitor (PDGFR, FLT3, RET, KIT, VEGFR1/2, CSF1R)	Phase II/III (recurrent glioblastoma)
Aflibercept [66]	VEGF-trap fusion protein	Phase II
Cediranib [67], [68]	VEGF receptor tyrosine kinase inhibitor	Phase II/III
Apatinib [69]	VEGFR-2 inhibitor	Preclinical
Sorafenib [70]	Multi-kinase inhibitor (VEGFR, PDGFR, RAF)	Phase I

### Clinical trial study design idea

In a potential Phase I/II clinical trial, a Phase I dose-escalation stage based on a 3 + 3 design may be planned primarily to determine safety and tolerable dose. In this phase, it can be evaluated whether the HIF inhibitor and VEGF inhibitor can be administered sequentially or concurrently with greater safety. In the Phase II phase, the determined optimal combination dose should be tested for efficacy in patient subgroups based on biomarkers such as 18 F-FMISO PET hypoxia positivity and HIF/VEGF overexpression. The primary endpoint at this stage could be PFS or objective response rate (ORR), while the secondary endpoints could be overall OS, biomarker response, and safety profile. Such a study design would increase the translational value in evaluating the clinical applicability of the combination.

### Combination ordering and biological rationale

In the first step, administration of an HIF-2 inhibitor suppresses the HIF-1/2-mediated hypoxia response in the TME; this reduces the cells’ capacity to repair radiation damage and hypoxia-induced radioresistance. Subsequent radiotherapy then induces more effective tumor cell death in a tumor with relatively increased oxygenation and limited adaptive response mechanisms (HIF inhibition increases radiosensitivity by preventing the HIF-1-induced VEGF increase that occurs during RT). The VEGFR2 inhibitor administered in the final stage blocks the angiogenic recovery process induced by radiation; thus, the remaining tumor cells after RT are prevented from forming new vessels and feeding themselves, and edema and tissue damage are controlled. The biological rationale for this sequential approach is to prevent the use of HIF pathway activation as a tumor escape mechanism both before RT (hypoxic protection) and after RT (angiogenic repair). Indeed, preclinical studies have shown that HIF-1 inhibition renders tumor cells in a hypoxic environment more sensitive to RT, and that suppressing the Notch-HIF-1 upregulation induced by RT with inhibitors produces a synergistic effect. Therefore, administering HIF inhibitors before RT and targeting the VEGF axis after RT is proposed as a logical and potentially beneficial strategy based on current data.

### Side effects of HIF inhibitors

HIF-2α inhibitors have a side effect profile due to the pathway they target. Anemia and tissue hypoxia especially have been defined as on-target toxicities of these agents [71, 72]. HIF-2α inhibitors such as Belzutifan may cause a decrease in hemoglobin levels and a reduction in tissue oxygen saturation by reducing erythropoietin production in the kidneys and affecting the oxygen sensing mechanisms in the lungs. As a matter of fact, the most common adverse effect observed in belzutifan studies for RCC was anemia, occurring in up to 90% of cases; during the clinical development process, patients requiring dose reduction, treatment discontinuation, or supplemental oxygen due to signs of hypoxia have been reported. In glioblastoma patients, anemia and related symptoms were also observed in > 60% of cases during belzutifan treatment. Therefore, from a pharmacovigilance perspective, regular monitoring of hemoglobin and oxygen saturation should be performed in patients treated with HIF inhibitors. If grade ≥ 3 anemia develops during treatment or if the patient experiences hypoxia symptoms such as palpitations, shortness of breath and confusion, dose reduction or temporary drug interruption may be necessary. Supportive measures such as blood transfusion and oxygen support should be taken if necessary. The risk of cardiotoxicity may be related to these agents indirectly reducing oxygen delivery rather than causing direct myocardial damage. Particular attention should be paid to the potential for anemia/hypoxia induced by belzutifan to lead to myocardial ischemia or progression of congestive heart failure, especially in elderly patients and those with low cardiopulmonary reserve. In this patient group, the drug’s dose and performance status should be closely monitored, treatment should be started with lower doses if necessary, and it should be remembered that tissue oxygen sensitivity increases with advancing age. Similarly, in treatment with the VEGF inhibitor bevacizumab; hypertension, risk of thromboembolic events, and rarely cardiac dysfunction may occur; therefore, monitoring of blood pressure and cardiac function is recommended, especially in combination therapies.

## Discussion

Although VEGF inhibitors have not demonstrated a significant improvement in OS among glioblastoma patients, future studies may yield different outcomes. This is supported by the critical role of VEGFR2-mediated signaling in glioblastoma pathophysiology, suggesting that targeted inhibition of this pathway remains a promising strategy for enhancing therapeutic efficacy and improving OS [5]. However, achieving this goal will require extensive intratumoral investigations. It is important to note that RT has been shown to induce activation of HIFs across multiple cancer types, not limited to glioblastoma. Upon HIF activation, there is a consequent upregulation of VEGF expression, which contributes to enhanced angiogenesis [48]. Adding a HIF-2α inhibitor to standard treatment in glioblastoma prior to RT may reduce the proportion of HIF activated by RT or inhibit this activation. While clinical trials have suggested that the sequential administration of VEGF inhibitors following HIF inhibition may lead to improved OS, further evidence is needed to validate this therapeutic approach. To date, none of the clinical trials with VEGF inhibitors used HIF inhibitors, but RT was always used and HIF pathways actively increased VEGF expression in glioblastoma. Similarly, clinical trials with HIF inhibitors in glioblastoma patients did not use VEGF inhibitors. Accordingly, adding VEGFA inhibitors or multi-kinase inhibitors such as Sunitinib simultaneously with HIF inhibitor in clinical trials may well suppress angiogenesis of glioblastoma, slow tumor progression, and achieve improved OS. Patients with signs of tumor hypoxia detected by advanced imaging such as ^18^F-FMISO PET or oxygen-sensitive biomarkers may be prioritized for evaluation for combined VEGF/HIF inhibition treatment and may be especially included in clinical trials based on criteria classification [16]. Although preclinical studies and known mechanisms suggest that this approach has synergistic anti-tumor effects, the lack of human trial data indicates that this combination treatment is currently speculative and unmet need exists.

In terms of side effects, hematological and metabolic toxicities such as anemia, tissue hypoxia, fatigue, and hyperglycemia have been reported, especially with the use of HIF-2α inhibitors [71, 73]. In addition, cardiovascular complications, especially the risk of developing heart failure, have been reported with long-term use. Therefore, the benefit-risk balance of combination treatment is carefully evaluated; however, given the low OS and PFS in glioblastoma patients, it may be more open to clinical trials compared to other indications. However, the risk of toxicity may be higher in elderly patient populations with high sensitivity to hypoxia or in groups with concomitant cardiovascular disease; glioblastoma patients are also typically diagnosed at an average age of 54 years. It is especially important in the elderly glioblastoma patient population that future clinical trials comprehensively evaluate and manage not only efficacy but also the safety profile.

The interaction between the HIF signaling pathway, VEGF-mediated angiogenesis, and the tumor’s response to RT is schematically illustrated. Stabilization of HIF-1α/2α in the hypoxic TME induces the overexpression of genes such as VEGF, FGF, and EPO; thereby supporting angiogenesis, metabolic adaptation, and cell survival. RT induces cell death in tumor cells via DNA damage and oxidative stress; however, the angiogenic and anti-apoptotic responses that develop in the presence of active HIF help tumor cells survive radiation. HIF-2α inhibitors such as PT2385 and belzutifan aim to halt these adaptive responses by preventing HIF-α dimerization with ARNT, while VEGF inhibitors correct tumor oxygenation by suppressing abnormal vascularization [74–76]. This combination strategy aims to overcome hypoxia-induced radiation resistance and enhance the efficacy of RT. Additionally; it has been demonstrated that hypoxic stress following bevacizumab enhances HIF-1-dependent cellular protective responses. Hu et al. reported that when bevacizumab was administered alone in glioblastoma xenografts HIF-1α-dependent BNIP3 levels increased and autophagy was activated, promoting tumor growth; this adaptive response could be prevented by an autophagy inhibitor such as chloroquine [77]. These results support our idea that hypoxia induced by anti-VEGF treatment promotes survival via HIF-1 and that targeting HIF-1 could enhance the efficacy of combination treatment by counteracting resistance development. Also, since HIF1A regulates the AKT and PDGFs pathways that support glioblastoma development, therapies that directly target HIF-1α alone show meaningful antitumor effects. Peng and colleagues demonstrated that silencing the HIF1A gene in orthotopic GBM models inhibited tumor growth and prolonged mouse survival, while the HIF-1α inhibitor Echinomycin suppressed the HIF1α-PDGFD/PDGFRa-AKT axis in GBM cells, increased apoptosis, stopped tumor progression, and increased survival in mice [78]. However, PDGFD overexpression in GL261 can reduce this survival. Such results support the idea that simultaneously targeting HIF-1α and other targets could be a powerful strategy to enhance the efficacy of anti-VEGF therapies.

## Conclusions

The lack of improvement in OS with VEGF inhibitors in glioblastoma patients has been attributed to RT-induced hypoxia-related mechanisms that sustain angiogenesis, and tumor progression. RT, one of the standard therapies for glioblastoma treatment, exacerbates HIF-mediated resistance by inducing VEGF expression. To date, clinical trials investigating VEGF inhibitors have not incorporated HIF inhibitors, and conversely, trials evaluating HIF inhibitors have not too included VEGF-targeted agents. The unmet need due to the lack of research in these combinations limits our understanding of their potential synergistic effects in glioblastoma treatment. Given the critical roles of both VEGF and HIF signaling pathways in glioblastoma biology, future research should prioritize on the testing of combination treatments that concurrently target these mechanisms to enhance therapeutic efficacy. Treatment with HIF inhibitors prior to RT may reduce the HIF activation caused by RT, thereby enhancing the efficacy of subsequent VEGF-targeted therapies. Addressing this therapeutic gap through rigorously designed clinical trials could inform the development of novel treatment protocols aimed at improving survival outcomes in patients with glioblastoma.

## Data Availability

No datasets were generated or analysed during the current study.
